# Pathways for Processing Noise: Heart Health and the Sounds of Everyday Life

**DOI:** 10.1289/ehp.121-a167

**Published:** 2013-05-01

**Authors:** Julia R. Barrett

**Affiliations:** Julia R. Barrett, MS, ELS, a Madison, WI–based science writer and editor, has written for *EHP* since 1996. She is a member of the National Association of Science Writers and the Board of Editors in the Life Sciences.

Epidemiological studies suggest that noise exposure affects cardiovascular system function. Most studies have focused on links between specific types of chronic loud noises associated with jobsites or roadways and adverse cardiovascular effects including high blood pressure and heart disease. But the biological underpinnings of these relationships have been little explored, particularly with regard to noise encountered during everyday life. A new study finds that lower-intensity everyday noises also can affect the cardiovascular system, although the effects are likely mediated via different pathways than those associated with effects of louder noise [*EHP* 121(5):607–612; http://dx.doi.org/10.1289/ehp.1205606].

Environmental stimuli influence the cardiovascular system via the autonomic nervous system, which regulates involuntary body functions such as respiration and heartbeat. Noise impacts can be assessed by measuring heart rate and heart-rate variability (HRV). HRV is influenced by sympathetic and parasympathetic activity, which may be considered the accelerator and brakes, respectively, of the autonomic nervous system. These pathways balance each other to ensure optimal heart function, and changes such as reduced HRV have previously been associated with increased risk for adverse cardiovascular effects.

The current study took place in Augsburg, Germany, between March 2007 and December 2008, as part of a larger series of investigations by the Rochester Particulate Matter Center. Participants provided their medical history and health information at the start of the study; during its course they completed up to four examinations with individual exposure measurements. For the examinations, which averaged 6 hours, 110 participants wore electrocardiogram equipment that tracked heart-related variables and dosimeters that measured noise and air pollutant (ultrafine particulate matter) exposures as they undertook their normal daily activities.

**Figure:**
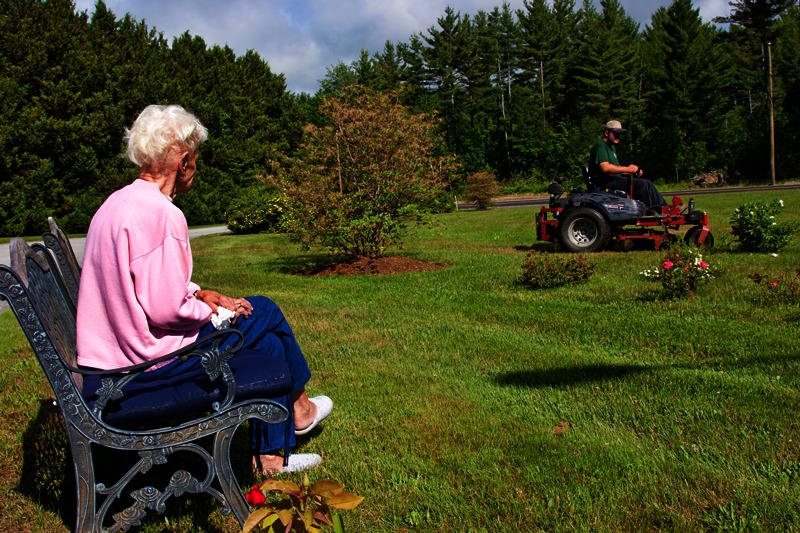
Both high- and low-intensity noises can affect the cardiovascular system but appear to do so by different mechanisms. © Vicki Beaver/Alamy

For data analysis, the researchers categorized noise exposures as low-intensity or high-intensity, defined by a cutoff point of 65 dB(A), a weighted measure of perceived loudness. Within these categories, the percent changes in HRV parameters were assessed for each 5-dB(A) increase in noise intensity. Increases within the low-intensity category appeared associated with reduced parasympathetic activity and possibly an increase in sympathetic activity. With increases within the high-intensity category, both sympathetic and parasympathetic activity seemed to increase, but net effects were consistent with the former exceeding the latter. Although these differing responses point to separate underlying mechanisms, both could ultimately reduce HRV and lead to increased cardiovascular risk.

The study included a large number of individual-level measurements, control for air pollution effects and physical activity, and separate consideration of low- and high-intensity noise. There may have been residual confounding and data gaps from unmeasured variables, and because the study population was made up of older adults, conclusions may not extend to the general population. Nevertheless, the findings suggest that low-intensity noise activates biological pathways separate from the “fight-or-flight” response triggered by louder noises; the potential health consequences should be explored.

